# Prevalence, Distribution and Functional Significance of the −237C to T Polymorphism in the *IL-12Rβ2* Promoter in Indian Tuberculosis Patients

**DOI:** 10.1371/journal.pone.0034355

**Published:** 2012-04-03

**Authors:** Vikas Kumar Verma, Vibha Taneja, Anand Jaiswal, Sangeeta Sharma, Digamber Behera, Vishnubhatla Sreenivas, Shyam Singh Chauhan, Hanumanthappa Krishna Prasad

**Affiliations:** 1 Department of Biotechnology, All India Institute of Medical Sciences, Ansari Nagar, New Delhi, India; 2 LRS Institute of Tuberculosis and Respiratory Diseases, New Delhi, India; 3 Department of Biostatistics, All India Institute of Medical Sciences, Ansari Nagar, New Delhi, India; 4 Department of Biochemistry, All India Institute of Medical Sciences, Ansari Nagar, New Delhi, India; International Center for Genetic Engineering and Biotechnology, India

## Abstract

Cytokine/cytokine receptor gene polymorphisms related to structure/expression could impact immune response. Hence, the −237 polymorphic site in the 5′ promoter region of the *IL-12Rβ2* (SNP ID: rs11810249) gene associated with the AP-4 transcription motif GAGCTG, was examined. Amplicons encompassing the polymorphism were generated from 46 pulmonary tuberculosis patients, 35 family contacts and 28 miscellaneous volunteers and sequenced. The C allele predominated among patients, (93.4%, 43/46), and in all volunteers and contacts screened, but the T allele was exclusively limited to patients, (6.5%, 3/46). The functional impact of this polymorphism on transcriptional activity was assessed by Luciferase-reporter and electrophoretic mobility shift assays (EMSA). Luciferase-reporter assays showed a significant reduction in transcriptional efficiency with T compared to C allele. The reduction in transcriptional efficiency with the T allele construct (pGIL-12Rb2-T), in U-87MG, THP-1 and Jurkat cell lines, were 53, 37.6, and 49.8% respectively, compared to the C allele construct (pGIL-12Rb2-C). Similarly, densitometric analysis of the EMSA assay showed reduced binding of the AP-4 transcription factor, to T compared to the C nucleotide probe. Reduced mRNA expression in all patients (3/3) harboring the T allele was seen, whereas individuals with the C allele exhibited high mRNA expression (17/25; 68%, *p* = 0.05). These observations were in agreement with the *in vitro* assessment of the promoter activity by Luciferase-reporter and EMSA assays. The reduced expression of IL-12Rβ2 transcripts in 8 patients despite having the C allele was attributed to the predominant over expression of the suppressors (IL-4 and GATA-3) and reduced expression of enhancers (IFN-α) of IL-12Rβ2 transcripts. The 17 high IL-12Rβ2 mRNA expressers had significantly elevated IFN-α mRNA levels compared to low expressers and volunteers. Notwithstanding the presence of high levels of IL-12Rβ2 mRNA in these patients elevated IFN-α expression could modulate their immune responses to *Mycobacterium tuberculosis*.

## Introduction

Approximately 10% of the individuals, exposed to *Mycobacterium tuberculosis*, develop clinical disease [1], the remaining individuals are able to restrict, and eliminate the infection by generating an appropriate immune response. Accumulating evidence suggests that genetic factors contribute to variations in host response against *M. tuberculosis*. Hence, the variety of cytokines expressed during the incubation period critically influences the quality and the dominance of the type of immune response, [2]. Therefore, polymorphisms in cytokine genes and their receptors that could potentially modify expression and or biological activity would be of particular interest, [3]. Polymorphisms in the genes of cytokines and their receptors have been reported to be associated with tuberculosis disease status in various populations, [4].

Polymorphisms in the regulatory regions of the genes have been associated with variation in gene expression, [5]. The present study focuses on the investigation of the functional role of polymorphism in the promoter region of the *IL-12Rβ2* gene. The heterodimeric IL-12 receptor consists of IL-12Rβ1 and β2 subunits. The β2 chain along with β1 chain constitutes the high affinity IL-12 binding site. The effectiveness of IL-12 biological function is determined by the presence of the IL-12 receptors on the cells. Both receptor subunits bind IL-12; however the signal transducing component is exclusively limited to the IL-12Rβ2 chain, [6]. Further, the IL-12Rβ2 chain is restricted in its distribution among Th1 cells, [7], [8]. Several transcription factors such as SP-1, SP-3, NFATc2, GATA- 3, Oct-1, etc., regulate expression of the *IL-12Rβ2* gene [9], [10]. The alteration in the promoter activity of *IL-12Rβ2* gene has been reported with the base exchange at the following sites namely −1110, −1035, −628, −890 and −465. Besides these sites, the polymorphism at the −237 position, (SNP ID: rs11810249) has been reported previously in asthma [10]. As predicted by *in silico* analysis, the −237 polymorphic site is a part of the AP-4 transcription factor binding motif. AP-4 is a ubiquitously expressed transcription factor, which belongs to the basic helix-loop-helix leucine zipper (hHLH-LZ) subgroup of bHLH proteins and recognizes the symmetrical DNA core sequence CAGCTG [11]. This motif has been referred throughout this manuscript as the consensus motif. In the *IL-12Rβ2* promoter region the motif is predicted to be located at position −234 to −239. This predicted cognate motif has a conserved substitution at position −234 C to G. This sequence namely GAGCTG has been referred to as the *IL-12Rβ2* AP-4 motif. As the −237 polymorphism is located in the regulatory region, this may potentially modulate the *IL-12Rβ2* gene expression which in turn may influence the biological activity of IL-12 required in the genesis of host immune response to *M. tuberculosis*.

Apart from examining the role of the polymorphism at the −237 site related to IL-12Rβ2 mRNA expression, cytokines/transcription factors that are known to be associated with enhancement/suppression of IL-12Rβ2 mRNA expression have been investigated. The suppressors included were the cytokine IL-4, [7] and the transcriptional factor GATA-3, [12]; and the enhancer considered was IFN-α, [13].

## Results

**Figure 1:**
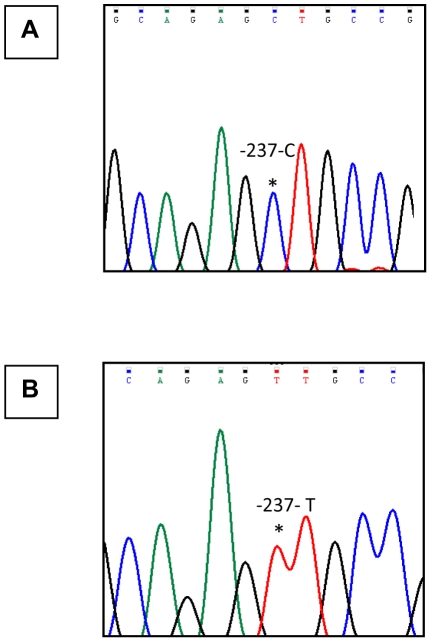
PCR amplification and sequencing analysis chromatograms for the −237C/T polymorphism. **Panel (A & B):** Chromatogram showing the sequencing analysis of the 622 bp amplicon derived from individual with polymorphic C (shown in panel A), or T type alleles (shown in panel B) respectively. The presence of the wild type −237C and the polymorphic −237T allele has been indicated with (*).

The one of the objectives of the present study was to detect the presence of the −237 C/T polymorphism (SNP ID: rs11810249) in the *IL-12Rβ2* promoter region and to assess its distribution among tuberculosis patients, household contacts and miscellaneous healthy volunteers. For this purpose, we subjected the 622 bp DNA amplicons encompassing the polymorphic site, derived from 109 individuals to double stranded DNA sequencing. The results of this analysis are presented in Figure 1 & Table 1. Examining the polymorphic position −237 C/T, it was seen that the C_−237_ site was present in 93.4% (43 / 46) patients and in all contacts (35 / 35) & healthy volunteers (28 /28); whereas the T_−237_ position, was detected exclusively in 3 of the 46 (6.5%) tuberculosis patients.

**Table 1:** The distribution of −237 C/T polymorphism among patients, healthy contacts and volunteers.

Subjects^a^	−237 C/T^b^			
	C^c^		T^d^	
	N^e^	%^f^	N	%
**Patients** g	46	93.4	3	**6.5**
**Contacts** h	35	100	0	**0**
**Volunteers** i	28	100	0	**0**

^**a**^ Subjects included in the study;

^**b**^ Polymorphism position ;

^**c**^ Individuals with C nucleotide at −237 position;

^**d**^ Individuals with T nucleotide at −237 position ;

^**e**^ Total number of individuals investigated;

^**f**^ Percentage of individuals.

^**g**^ pulmonary tuberculosis patients ;

^**h**^ healthy patients contacts ;

^**i**^ Healthy laboratory Volunteers.

### Altered transcriptional activity

**Figure 2:**
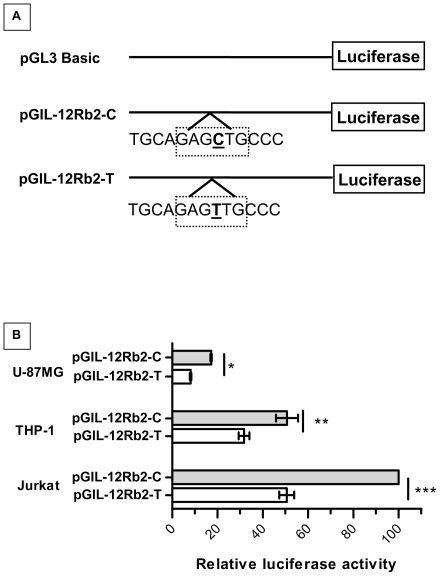
Schematic representation of the vector constructs and the luciferase activity observed in different cell lines. **Panel A**. Schematic representation of the promoter constructs, namely pGIL-12Rb2-C (with C allele) and pGIL-12Rb2-T (with T allele) that have been used for the luciferase reporter assays. The region extending from −591 up to +55 of the 5′ region the IL-12Rβ2 promoter was cloned into pGL3-Basic vector. The location of the polymorphic allele at −237 positions has been underlined. The dotted box indicates the AP-4 binding motif in the *IL-12Rβ2* promoter. **Panel B**: The vector constructs pGIL-12Rb2-C, pGIL-12Rb2-T and the pGL3-Basic vector (Negative control) were co-transfected along with pRL-TK vector into U-87, THP-1 and Jurkat cell lines as described in methods. The renilla activity expressed by the pRL-TK vector was used to normalize the transfection efficiency. The reporter gene Firefly luciferase activity was determined for each sample in triplicates, 42 hours post transfection. The relative luciferase activity was expressed as fold increase in the activity compared to the promoter less pGL3-Basic vector and the results has been shown as mean ± SEM of three independent experiments. Student's *t* test with unequal variances was carried out to compare the transcriptional efficiency (*) of the constructs, (*p = 0.0031; **p = 0.0097, ***p = 0.0001).

The substitution of T for C at the −237 polymorphic site alters the *IL-12Rβ2* AP-4 transcription factor binding motif GAGCTG, [10], [11], [14]. Therefore to evaluate the impact of C to T polymorphism on transcriptional efficiency, promoter-reporter constructs harboring the polymorphic binding sites were transfected into U-87MG, THP-1 and Jurkat cell lines, (Figure 2, Panel A). Figure 2, Panel B, shows the estimated relative luciferase activity with each of the constructs in the respective cell lines. Luciferase reporter gene driven by *IL-12Rβ2* promoter containing the C allele (pGIL-12Rb2-C) exhibited significantly higher luciferase activity in all cell lines compared to the T allele construct (pGIL-12Rb2-T). The maximum luciferase activity of 37.8±2.8 (Mean ± SEM) fold over the promoter less PGL3-Basic vector was observed in the Jurkat cell line with the construct pGIL-12Rb2-C. This maximal activity was set as a reference value of 100%, and promoter activity of other constructs was compared against this reference value. Accordingly, the activity of pGIL-12Rb2-T in Jurkat cell line was observed to be 50.6±3.3%. While the transcriptional activity of pGIL-12Rb2-C construct in THP-1 and U-87 MG cell lines was 50.7±4.9 and 17.2±0.3%, compared to the pGIL-12Rb2-T construct wherein it was reduced to 31.8±2.4 and 8.1±0.4% respectively.

The luciferase activity of the pGIL-12Rb2-T construct was lower in all the three cell lines investigated, (Figure 2, Panel B). The observed reduction in luciferase activity with the T compared to the C allele, in U-87MG, THP-1 and Jurkat cell lines was 52.7, 37.3 and 49.1% respectively. This reduction was statistically significant in the three cell lines examined, (*p* = 0.0031 for U-87 MG; *p* = 0.0097 for THP-1; *p* = 0.0001 for Jurkat cell lines respectively; Figure 2, Panel B). These results suggest the association of T allele with reduced promoter activity.

### C to T polymorphism alters AP-4 binding

**Figure 3:**
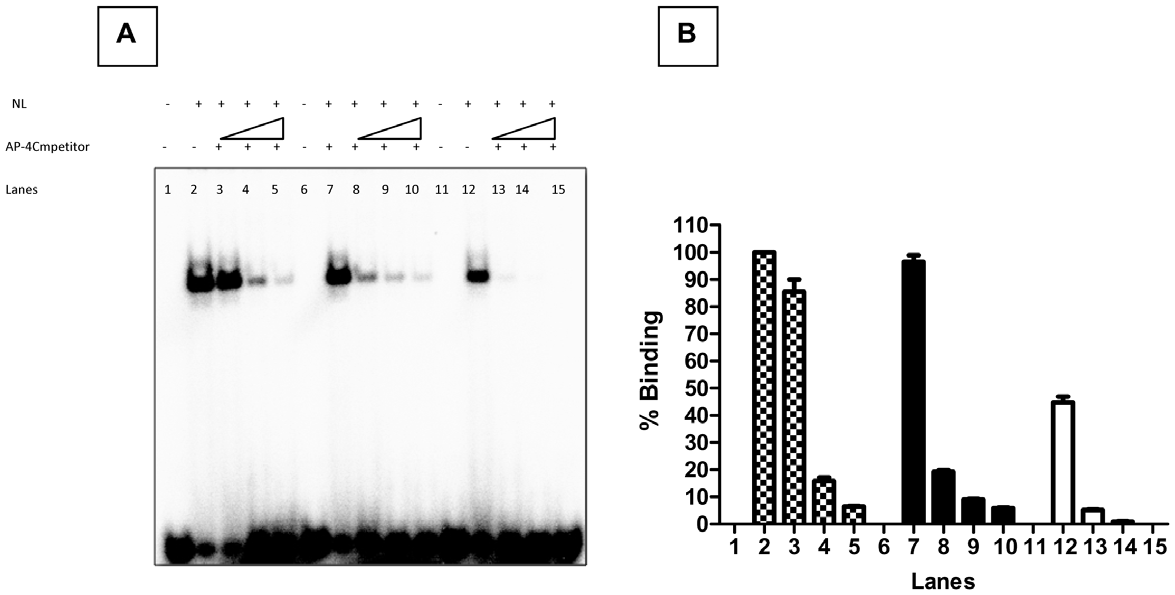
EMSA experiments for the assessment of AP-4 / C type / T type probe binding ability on addition of unlabelled AP-4 consensus oligos as competitor. **Panel (A)**: Shows the EMSA experiments carried out to assess the displacement of the probe complexed with the protein in the presence of excess molar concentrations (10, 50 & 100×) of the unlabelled AP-4 oligonucleotide as indicated. The probe-protein complex was generated using the labeled AP-4 consensus (Lanes 1–5), C (Lanes 6–10) and T probes (Lanes 11–15). **Panel (B)**: The bar diagram shows the densitometric profile of the autoradiograph depicted in Figure 2, Panel c. The labeled AP-4 consensus (Lanes 1–5), C probe (Lanes 6–10) and T probe (Lanes 11–15) were incubated with the nuclear lysate. The bars represent the mean ± SD of three experiments.

**Figure 4:**
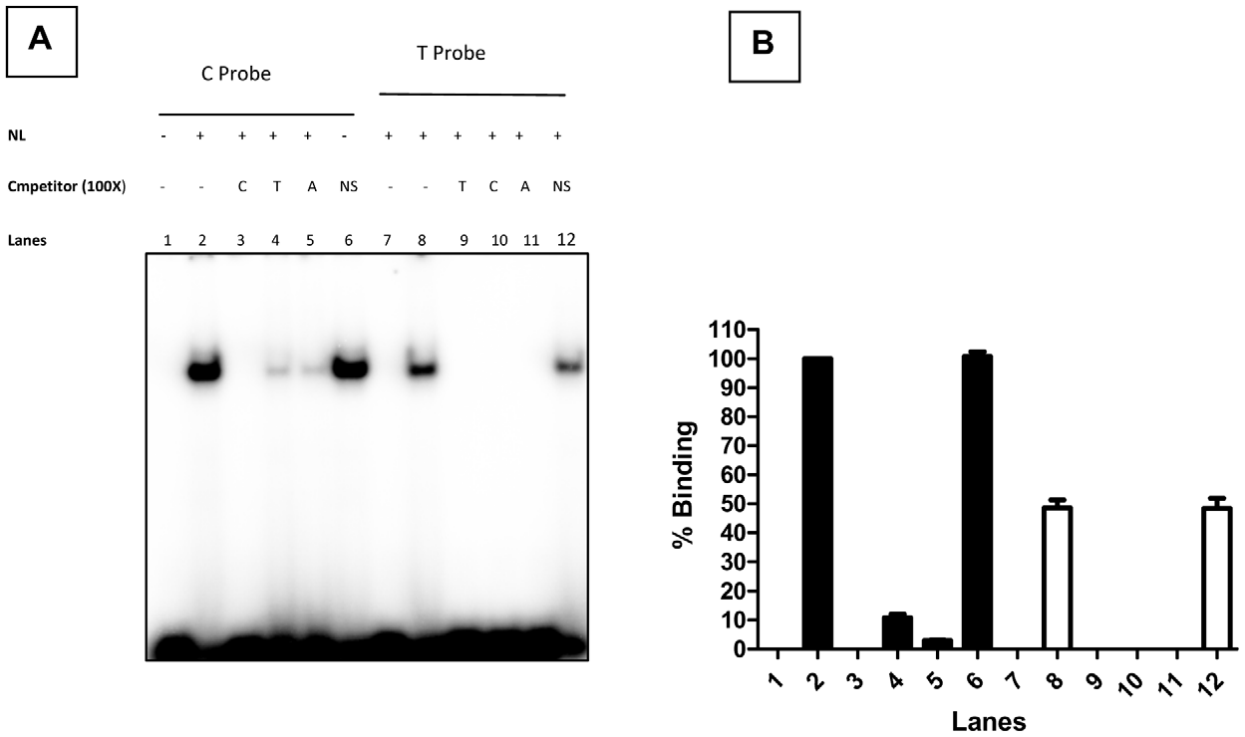
EMSA experiments to assess binding ability and specificity of probes. **Panel (A)**: Shows the EMSA experiments to evaluate the specificity of the Oligo – Nuclear protein complex formed with the C and T probes. The Oligo–Nuclear protein complex was formed in the presence of 100× excess molar concentration of the unlabelled competitors namely Self / Heterologous / AP-4 consensus / Non specific (CEBP-α, NS) oligos. **Panel (B)**: Bar diagram showing the densitometric profile of the autoradiograph, Figure 5.7, Panel A. The C & T type of oligo without nuclear lysate (Lanes 1 & 7) ; with nuclear lysate and without competitors (Lanes 2 & 8); with self competitors (Lanes 3 & 9) ; with heterologous competitors (Lanes 4 & 10 ); with unlabelled AP-4 consensus oligo ( Lanes 5 & 11) and with unlabelled non specific oligo (NS, CEBP-α, Lanes 6 & 12). The bars represent the mean ± SD of three experiments.

**Figure 5:**
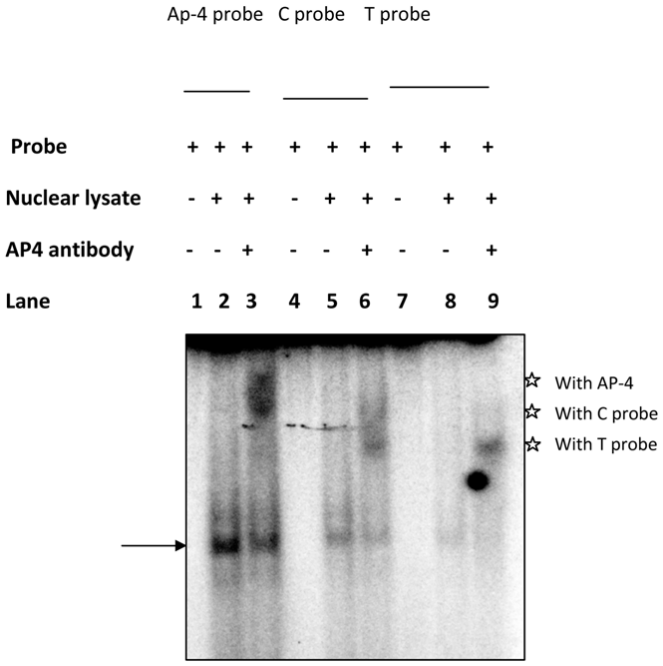
Supershift assay using AP-4 consensus, wild type and polymorphic type probe incubated with / without AP-4 polyclonal antibody. The assay carried out utilizing different probes without nuclear lysate ; (lanes 1 , 4 & 7); with Jurkat nuclear lysate ; (Lanes 2,5 & 8) Super shift assay with Jurkat cell nuclear extracts was carried out using anti AP-4 antibody (20 µl ) with specific AP- 4 probe (lane 3), C and T type probes, lanes 6 & 9 respectively. The arrow head indicates the shifted band corresponding to the different type of probes. The star symbol indicates supershifted complex corresponding to different probes. These data are representative of 2 independent experiments.

*In silico* analysis, revealed that C to T polymorphism, which caused a reduction in promoter activity, also abolishes the AP-4 binding to *IL-12Rβ2* AP-4 motif (GAGCTG). We employed EMSA to assess, the consequence of this polymorphism on binding of AP-4 transcription factor and the results are presented in Figures 3, 4, 5.

To establish and confirm the binding of C and T ds-oligos with the AP-4 transcription factor, the AP-4 consensus probe was used as a positive control. The binding efficiencies of C, T and AP-4 type probes to the nuclear factor was assessed in the presence of varying concentrations (0 to 100 molar excess) of the unlabeled ds-oligos containing the AP-4 consensus motif (CAGCTG, Figure 3, Panel A). All probes exhibited formation of a DNA-protein complex with the nuclear extract derived from PMA–PHA activated Jurkat cells, (Figure 3, Panel A, AP-4 probe Lanes 1–5; C probe Lanes 6–10; T probe Lanes 11–15). These complexes showed identical electrophoretic mobility suggesting the binding of similar nuclear factor(s) to the probes. Excess of unlabeled ds-oligos containing the consensus AP-4 binding motif could displace the radio labeled AP-4 probe from the DNA-protein complex. A similar displacement of the radio labeled C and T probes from their respective DNA-protein complexes by unlabeled AP-4 consensus ds-oligos was observed. These results suggest the specificity of the binding of the transcription factor AP-4, to the radio labeled AP-4, C and T probes.

Densitometric analysis of the DNA-protein complexes was carried out. The densitometric value of the AP-4 consensus probe-protein complex (positive control) was set as 100%, (Figure 4, Panel B, Lane 2). The analysis revealed comparable binding of AP-4 transcription factor to the AP-4 consensus and C-probes. Accordingly the degree of binding with C and T probes was estimated to be 96.5±2.3, (Mean ± SD, Lane 7) and 44.8±2.1% (Lane 12) respectively. Displacement assays revealed that a 10 molar excess of AP-4 consensus ds-oligo was sufficient to displace the T probe from the DNA-AP-4-protein complex. Whereas this displacement could not be achieved by 100 molar excess of the AP-4 ds-oligo in case of the AP-4 consensus or C probe complexes (Figure 3, Panel B, Lanes 5 & 10). These results revealed that the intensity of the DNA-protein complex with consensus AP-4 (100%) and C (96.5%) probes was comparable. However, the intensity of this complex with the T probe (44.8%) was approximately 2 fold lower (Figure 3, Panel B, Lanes 7 & 12).

No DNA-protein complex was detected when EMSA was carried out with the T probe in the presence of 100 molar excess of the unlabeled T or C ds-oligos (Figure 4, Panel A, Lane 9 & 10). However, 100 molar excess of unlabeled C ds-oligo (Lane3) but not T ds-oligo (Lane 4) could completely abolish the formation of this complex with the C probe. Densitometric analysis revealed that the T ds-oligo could abolish 90% binding of the C probe to AP-4 protein, (Figure 4, Panel B, Lane 4), whereas C ds-oligo could completely abolish the binding of the same factor with the T probe, (Figure 4, Panel B, Lane 10). The formation of the complex with T / C probe could not be abolished in the presence (100× molar concentration) of nonspecific competitor containing CEBP-α binding motif, (Figure 4, Panel A & B Lanes 6 & 12). These results further confirm the binding of AP-4 to C and T probes and the higher affinity of C compared to the T probe to AP-4 transcription factor.

In order, to identify the proteins binding to the polymorphic site, super shift experiments were conducted using the wild-type probe harboring the C nucleotide at −237 position and the polymorphic type probe harboring T, and nuclear extracts derived from Jurkat cells, (Figure 5). As this site previously has been predicted to bind AP-4 transcription factor, polyclonal antibody directed against transcription factor AP-4 was used in the super shift assay. Addition of antibody super shifted the labeled probe- lysate complex, (Figure 5, Lanes 3, 6, 9) compared to the complexes in the absence of the antibody, (Lanes 2, 5, 8). The shifted DNA- protein complex was further super-shifted maximally with AP-4 consensus probe (Figure 5, Lane 3) followed by the wild type C probe (Lane 6) and the polymorphic type T probe (Lane 9).

**Figure 6:**
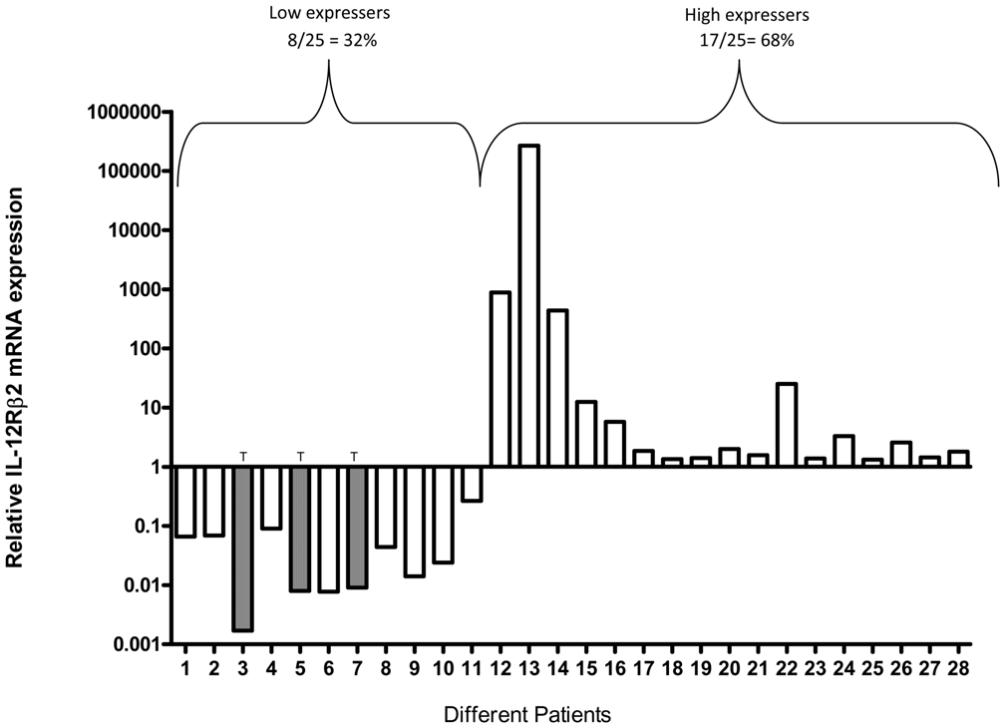
Relative mRNA quantification of IL-12Rβ2 mRNA expression in tuberculosis patients (N = 28). Histogram shows the IL-12Rβ2 mRNA expression profile in the blood of the tuberculosis patients. The identification of the polymorphism at −237 site has been carried out by PCR amplification and sequencing (forward & reverse) of the 622 bp region spanning −780 to −159. The patients with the T at −237 polymorphic site has been indicated. The bars represent expression of IL-12Rβ2 mRNA in each individual. The fold activity pattern in real time PCR assay was calculated as described in methods, [15]. The individuals with reduced / elevated IL-12Rβ2 mRNA expression have been designated as Low expressers / High expressers.

As IL-12Rβ2 surface expression was low in unstimulated circulating PBMCs (Data not shown), experiments were carried out to determine mRNA expression of the IL-12Rβ2 subunit at the baseline level in peripheral blood leucocytes derived from 28 tuberculosis patients, (Figure 6). Analysis was carried out to establish a relationship if any between the IL-12Rβ2 mRNA expression and the polymorphism at −237 position of the IL-12Rβ2 promoter.

### IL-12Rβ2 mRNA expression

The Relative transcript level (^_^ΔΔ Ct) was calculated after normalization with the β-actin mRNA and expressed with respect to that of healthy laboratory volunteers (N = 28) as described [15]. Individuals were categorized as high expressers when the deduced transcripts levels was >1, while individuals with transcripts levels <1 were categorized as low expressers. The mRNA expression of the 28 tuberculosis patients, investigated showed a mixed expression profile of the IL-12Rβ2 mRNA expression, (Figure 6).

The mRNA expression in 3 patients harboring the T allele at the −237 position was low, (<1; Figure 6, Patient numbers 3, 5 & 7 respectively). The remaining 25 patients harboring the C allele showed divergent expression of mRNA. 17 patients showed high levels of expression (>1; 68%) and the 8 patients showed low expression (<1; 32%). The high levels of expression of mRNA in the presence of C allele in 17 patients and the low levels of mRNA with the T allele in 3 individuals correlated with the *in vitro* functional assays related to promoter activity as observed in the luciferase reporter assays, (*p* = 0.05 ; Fisher's exact test). Similarly, the EMSA assays also confirmed the higher binding of the AP-4 transcription factor with the C type allele compared to the T allele.

### Assessment of IL-4, IFN-α and GATA-3 mRNA expression

In case of the 8 patients the IL-12Rβ2 mRNA expression was negligible despite their harboring C in the *IL-12Rβ2* AP-4 motif, which was in contradiction to the high transcriptional activity expected as determined by the reporter assay, (Figure 2). In this regard, experiments were designed to investigate if any, the possible role of additional modulators of IL-12Rβ2 expression such as IL-4, IFN-α and the master switch transcription factor GATA-3. IL-4 and GATA-3 suppresses, whereas IFN-α is a potent enhancer of IL-12Rβ2 expression, [7], [12], [13].

**Figure 7:**
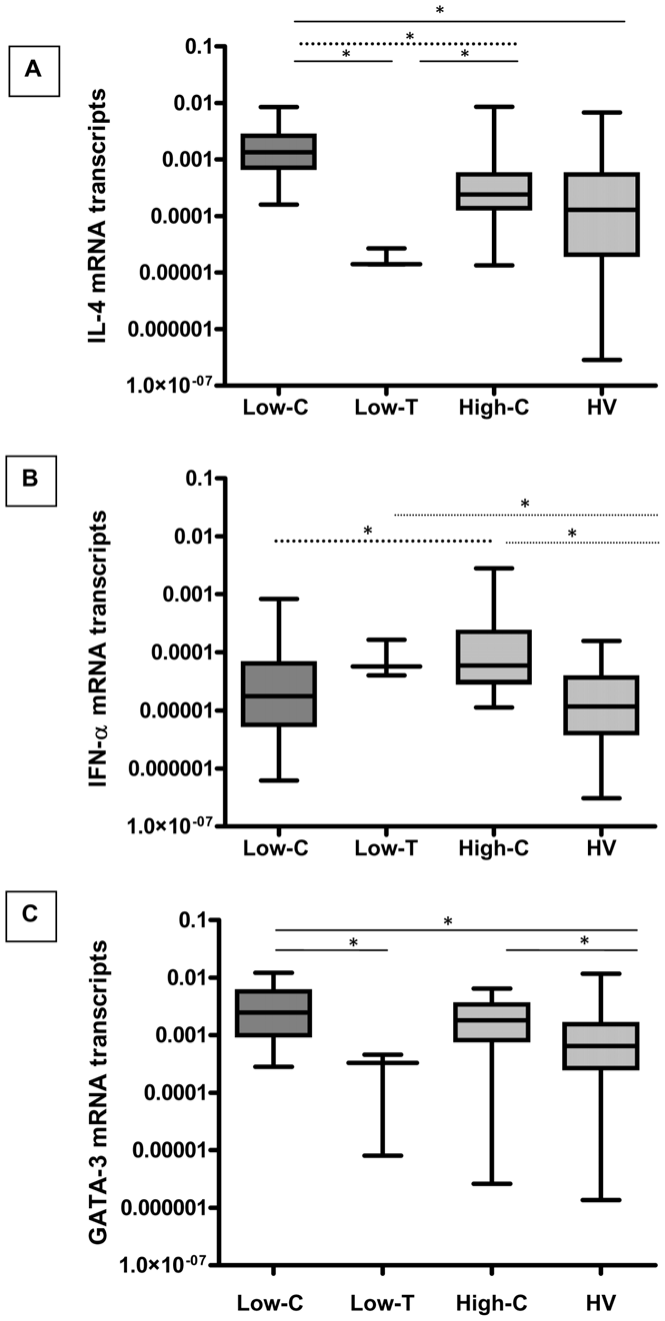
Real -Time PCR analysis for transcripts of suppressers (IL-4 and GATA-3) and enhancers (IFN-α) of IL-12Rβ2 mRNA in 28 tuberculosis patients and healthy volunteers. The Box plots represents the expression levels of **IL-4** (Panel A), **IFN-α** (Panel B) and **GATA-3** (Panel C) mRNA expression among the untreated Tuberculosis patients and healthy volunteers (HV), as estimated by Real-Time PCR. The patients were categorized based on IL-12Rβ2 mRNA levels detected and the presence of C/T allele at the −237 position, (Figure 6). Target gene expression was normalized with *β-actin* gene expression. The data has been calculated with the 2^−ΔCt^ formula, as described in methods. The horizontal bar represents the median value for mRNA in each group, the 25^th^ and 75^th^ percentile have been represented by the boxes. The whiskers represent the maximum and minimum values of the data, respectively. The data has been plotted on log_10_ scale. **Low-C**: Low IL-12Rβ2 mRNA expressers with allele C at −237 position (N = 8); **Low-T**: Low IL-12Rβ2 mRNA expressers with polymorphic allele T at −237 position (N = 3); **High–C**: High IL-12Rβ2 mRNA expressers with allele C at −237 position (N = 17); **HV**: Healthy volunteers free of tuberculosis (N = 28). To compare the transcript levels between groups, non-parametric Mann-Whitney Test was applied. (*) - Significant differences in transcript levels between compared groups have been indicated. **Panel A**: Low-C Vs Low-T, *p* = 0.01; Low-C Vs HV, *p* = 0.002; Low-C Vs High-C (⋅⋅⋅⋅⋅) *p* = 0.02; Low-T Vs High-C, *p* = 0.02;Low-T Vs HV & High-C Vs HV, not significant. **Panel B**: Low-C Vs High-C (⋅⋅⋅⋅), *p* = 0.04; High-C Vs HV, *p* = 0.002; Low-T Vs HV, *p* = 0.05. **Panel C**: Low-C Vs Low-T, *p* = 0.05; Low-C Vs HV, *p* = 0.02; High-C Vs HV, *p* = 0.04; Low-C Vs High-C, and Low-T Vs HV not significant.

The results of IL-4, GATA-3 and IFN-α mRNA expression among the 28 untreated tuberculosis patients and equivalent number of healthy volunteers have been depicted in figure 7. These patients have been categorized based on the levels of IL-12Rβ2 mRNA detected, into 17 high and 11 low expressers as described in Figure 6. In addition, the 11 low expressers were further sub-divided into 8 patients with the C allele (Low-C) and 3 with the T allele (Low-T) in the *IL-12Rβ2* AP-4 motif.

As a group healthy volunteers had lower transcript levels for all the three target genes investigated compared to tuberculosis patients, (IL-4, *p* = 0.03; IFN-α, *p* = 0.009 and GATA-3, *p* = 0.04). On the other hand, differences between the patient groups were seen. The 8 low expressers of IL-12Rβ2 mRNA (Low-C) had the highest transcript levels of IL-4 compared to the Low-T, (*p* = 0.01) & High-C (*p* = 0.02) group of tuberculosis patients and healthy volunteers examined, (*p* = 0.002, Figure 7, Panel A). On comparing the IFN-α transcripts, the Low-C patients had reduced transcript levels compared to the Low-T and High-C patient groups, (*p* = 0.04, Figure 7, Panel B). The highest GATA-3 transcript levels was seen among the Low-C compared to Low-T (*p* = 0.05) & High-C patients, besides healthy volunteers (*p* = 0.02, Panel C).

Patients with the T mutation (Low-T), showed low expression of IL-4 transcripts compared to Low-C (*p* = 0.01) & High-C (*p* = 0.02) patient groups and healthy volunteers, (Figure 7, Panel A). These patients showed higher IFN-α transcript's compared to Low-C & healthy volunteers and lower GATA-3 transcripts compared to Low-C, (*p* = 0.05) & High-C patients and healthy volunteers.

## Discussion

This is the first report of the analysis of the polymorphism −237C to T in the *IL-12Rβ2* gene and its association with tuberculosis. We found the presence of the T nucleotide at −237 position (SNP ID: rs11810249) of the *IL-12Rβ2* promoter exclusively in patients. Since the C/T−237 polymorphic site, lies on the putative AP-4 binding site, we investigated whether the difference in the IL-12 receptor expression is due to the variation in promoter activity owing to its differential binding. *In silico* analysis predicted the loss of the binding of the AP-4 transcription factor with C to T base exchange. The regulatory role played by the polymorphism namely C/T at the −237 position has been confirmed by luciferase reporter assays, wherein we found that the promoter construct with the C nucleotide had higher promoter activity compared to the T nucleotide promoter construct. In addition, the binding ability of C or T type probes with the AP-4 transcription factor was assessed by EMSA and super shift assays. The results showed that the C type of probe had greater affinity to bind the AP-4 transcription factor compared to the T type probe. This observation was in agreement with the promoter activity assessed by the luciferase reporter assays. In addition to *in vitro* assays, mRNA expression data showed that individuals with T allele had the lowest expression of mRNA compared to other counterparts. Hence polymorphism of C/T at −237 position could potentially affect the expression of the IL-12Rβ2 receptor component of the IL-12 receptor.

The factors that determine resistance or susceptibility to tuberculosis remain ill-defined, [2]. Polymorphisms both in the regulatory and coding region of the *IL-12Rβ2* gene have been identified in leprosy, (−1047delT, −1035A/G, −1033T>C,–1023A/G, −650delG, −568A/C, −557T/C, −550T/C, −464A/G −464A/C, 202T/C and−188A/C, [16] and in allergic, respiratory disorders such as asthma, (−1035A/G, −1033T/C, −1023A/G, −465A/G and −237C/T, [10]. Alterations in the promoter activity, associated with polymorphism in the genes of several cytokines and their receptors in tuberculosis, [17], [18] and other diseases have been examined, [19], [20].

In the present study, analysis of the polymorphic site showed that the T allele was found in 6.5% (3/46) and the C allele in 93.4% (43/46) of patients. Earlier reports on −237 polymorphism among Caucasoid asthmatics, the frequency of the T type allele in asthmatics was low (2.5%, 2/80) compared to the C allele. However in the study the distribution of −237 C/T in healthy controls and its functional consequence was not investigated, [10]. Mutations such as +1577A to G, and +2799 A to G and the truncated +2496 del 91, in the coding region of the *IL-12Rβ2* gene, have been associated with reduced IFN-γ production, [21].

As assessed by cross-competition experiments, the binding affinity to the protein(s) by the wild-type C allele variant was approximately 2-fold greater than that seen with the mutant T allele counterpart. The reduction in transcriptional efficiency in U-87MG, THP-1 and Jurkat cell lines was 53, 37.6 and 49.8% respectively. These results indicated the influential role played by the C/T nucleotide at −237 position for promoter activity of the *IL-12Rβ2* gene. The reduction in the promoter activity with the T allele was not tissue specific, however, the degree of reduction was tissue specific, where it was least reduced in THP-1 cell line. The highest transcriptional activity of the constructs (pGIL-12Rb2-C and pGIL-12Rb2-T) was observed in the Jurkat cell line followed by THP-1 and least in U-87MG cell line. The highest activity observed in Jurkat cells (human T cell lymphoma) may be due to the fact that expression of IL-12Rβ2 is primarily associated with Th1 type of T cells, [8], [22]. We predict that the higher activity of the promoter with the C allele and reduced activity with the T allele was due to the alteration in the binding of the transcription factor AP-4.

Super shift experiments confirmed the binding of AP-4 to the probes with C/T nucleotide at the −237 position. The probe-protein complexes formed with C/T probes migrated to the position identical to that seen with AP-4 consensus sequence. Complexes were further displaced upon addition of specific polyclonal antibodies. As the T allele showed comparatively less intensive binding as well as decreased transcriptional activity, it appears, *in vitro* AP-4 seems to function as an activator, in the *IL-12Rβ2* promoter. The change, in the AP-4 protein binding affinity at this polymorphic site, could be potentially responsible, for the alteration, in the *in vivo* expression of the *IL-12Rβ2* gene.

AP-4 has been described previously as an activator [14] as well as a repressor of gene transcription, [23], [24] indicating its probable regulatory role in gene expression. The role of polymorphism affecting the AP-4 binding has been assessed in *Pin1* promoter with relation to Alzheimer's disease, where the role of AP-4 as a transcriptional repressor has been reported in the context of G to C base exchange, [25]. In contrast, the functional investigation of the SNP at the AP-4 binding site at position −56 (T to C) in the promoter of *interferon-gamma receptor 1* (*IFNγR1*) gene in tuberculosis patients and other ethnic groups, showed loss of promoter activity, [18].

Role of potential GATA3 binding motifs present at positions −1035, −1110, −890, −628, and −465 of the *IL-12Rβ2* promoter on transcriptional efficiency have been previously assessed. The mutation at the −1035 site did not alter the binding, whereas mutation at the −465 position a threefold increase in transcriptional efficiency was seen. When the non-polymorphic GATA binding sites (−628, −890, −1110) were mutated no alteration in the transcriptional efficiency was seen [10]. The role of the promoter polymorphisms in other cytokine receptor genes, such as G-611A & T-56C of the *IFNGR1* gene showed no association with susceptibility to mycobacterial infection, [18]. However, an association between IL-10 promoter polymorphisms at −819 and −592 sites with elevated IL-10 levels in pleural tuberculosis has been reported, [17].

In 17 patients who harbored the C allele (17 / 25; 68%, High-C) high levels of IL-12Rβ2 mRNA expression was detected. On the other hand, despite the presence of C allele at the −237 site in 8 patients (Low-C) reduced levels of IL-12Rβ2 mRNA were detected. Comparing the suppressors (IL-4, GATA-3) and enhancer (IFN-α) of IL-12Rβ2 expression in these individuals a direct relationship was observed between the high and low expression of IL-12Rβ2 mRNA. In 17 patients, high transcripts of IFN-α and low levels of IL-4 & GATA-3 transcripts was detected, whereas in 8 patients with low levels of IL-12Rβ2 expression (Low-C), significantly high transcripts levels of IL-4 compared to all other groups of patients and healthy volunteers was observed. Besides high transcript levels of IL-4 & GATA-3 were detected, a concomitant reduction in IFN-α mRNA was detected in these 8 individuals. GATA-3 is a known to inhibit of the differentiation of TH1 lineage of T cells by suppressing the expression of the IL-12Rβ2 [12], whereas IFN-α inhibits GATA-3 expression, [26]. Hence, the appropriate conditions for IL-12Rβ2 mRNA expression was prevalent among the 17 high expressers and was absent in the 8 low expressers. Whereas the 3 patients (Low-T) with low levels of IL-12Rβ2 expression had equivalent transcript levels of IFN-α and low levels of IL-4, a condition conducive for optimal expression of IL-12Rβ2. However these individuals failed to express IL-12Rβ2 owing to the presence of the polymorphic allele T in the AP-4 motif.

Apart from IL-4 and GATA-3, other suppressors include IL-10 [17], [27], TGF-β [28] and IL-17 [29]. Additionally, the presence of stress indicators such as cortisols and prostaglandin E2 [30] could contribute to the depression of IL-12Rβ2 mRNA expression. High levels of these cytokines [17], [31] and corticosteroids [32] have been detected in serum/at the disease foci of tuberculosis patients. Further, *in vitro*, induction of IL-4 has been reported in patients following exposure to mycobacterial antigens, [33]. Additionally, high levels of circulating plasmacytoid dendritic cells a copious source of IFN-α have been reported to be present in untreated tuberculosis patients, [34] could account for the elevated IFN-α transcripts detected in patients in the present study. IFN-α is known to down regulate IFNGR1 receptor in infected macrophages, thus making macrophages intransient to IFN-γ induced activation, [35], [36]. We have reported the down regulation of IFNGR1 following infection with *M. tuberculosis*, [37]. Besides down regulation of IFNGR1 receptor, IFN-α inhibits the synthesis of the IL-12p40 subunit of the IL-12 cytokine by human antigen presenting cells, [36]. Hence elevated levels of IFN-α could be a pre-disposing factor for the onset of clinical tuberculosis despite its potency to induce expression of IL-12Rβ2 mRNA. Besides IFN-α, IFN-γ has been shown to upregulate the expression of IL-12Rβ2, [7], [8]. Elevated levels of IFN-γ have been reported in the disease site [38] as well as in circulation among tuberculosis patients, [33], [34].

The higher expression of the IL-12Rβ2 receptor in 17 patients did not result in resistance to *M. tuberculosis*. The plausible reasons could be that, the over expression of IL-12Rβ2 has been reported to sustain murine T regulatory (Treg) cells [39]. Higher levels of FOXP3 mRNA have been observed in tuberculosis patients, [40], [41]. FOXP3 protein is expressed exclusively in Treg cells, [42]. Increased transcription of *IL-12Rβ2* gene in patients could result in increased expression of the IL-12Rβ2 chain, in CD4+Tregs. The genesis and prevalence of Treg cells could initiate an inappropriate immune response(s) in patients leading to progressive disease.

Low IL-12Rβ1 expressing PBMCs has been detected in tuberculosis patients, [28]. Hence lack of expression of the IL-12Rβ1 chain on immune-competent cells despite the presence of IL-12Rβ2 chain would result in impairment in the binding of IL-12. As a consequence, would lead to the failure of the development of functional dendritic and NK cells, and critically impact the host immune response. However, both IL-12Rβ1 & β2 mRNA in bronchoalveolar lavage have been detected in tuberculosis patients, [43]. Besides the coordinated expression of both IL-12Rβ1 & β2 chains and the availability of the composite IL-12R in patients, yet another scenario exists wherein, the availability of IL-12 appears to be limiting, [34]. No one factor appears to be universal among the patients of tuberculosis that determines the loss of IL-12 bio-activity. The combination of factors that appear to be accountable for the disease is varied as the range of individuals susceptible to *M. tuberculosis*.

## Materials and Methods

### Ethics statement

The institutional ethical committees of the All India Institute of Medical Sciences (AIIMS), and Lala Ram Sarup Institute of Tuberculosis and Respiratory Diseases, Mehrauli, New Delhi, India, (LRS), approved the study. All individuals included in the study were Informed about the study and written consent was obtained from them.

### Study subjects

A total of 109 individuals consisting of untreated pulmonary tuberculosis patients who were sputum AFB positive (N = 46; Mean age: 31 years; 26 males and 20 females) registered in the Out-patient Department (OPD) of the LRS hospital and healthy family contacts of the patients (N = 35; Mean age 31.8 years; 20 males and 15 females) who were related / closely associated with the patients were included in the study. Miscellaneous healthy laboratory volunteers (N = 28; Mean age: 30 years; 18 males and 10 females) from Department of Biotechnology, AIIMS, were included as controls. All patients underwent clinical examination, chest X-ray, sputum microscopy for acid fast bacilli (AFB), and routine laboratory tests. Diagnosis of tuberculosis was made on the basis of clinical and radiological assessment and detection of AFB in sputum. Scrutinizing clinical histories of patients, physical examination, and laboratory investigations ruled out the occurrence of concomitant intracellular infections, in the enrolled individuals. All patients were human immunodeficiency virus negative. The healthy family contacts and miscellaneous healthy laboratory volunteers were screened for clinical signs of tuberculosis and were radiologically examined; when warranted on the basis of the symptoms, additional tests such as sputum examination for AFB and erythrocyte sedimentation rate were undertaken.

### Genomic DNA isolation

DNA was extracted from 300 µl of acetate citrate dextrose (ACD) collected blood by using Wizard Genomic DNA isolation kit (Promega, MD, USA) according to the manufacturer's instructions. The PCR reaction mixture in a final volume of 20 µl contained, 1× PCR buffer, 200 µM dNTPs, 2.5 mM MgCl2, 0.5 µM of each primer IL-12F-780 and IL-12R -159 with 1.0 U of Taq DNA polymerase (MBI Fermentas, Lithuania) and 100 ng of genomic DNA as template. A negative control without DNA was also included. Initial denaturation of the reaction mixture at 95°C for 5 min, and 35 cycles of each denaturation (94°C, 45 sec), annealing (62°C, 45 sec) extension (72°C, 45 sec) and a final extension at 72°C for 10 min, was carried out.

### Detection of amplified products and sequencing analysis

The resulting PCR amplified products (622 bp) were analyzed by agarose gel electrophoresis (1.5%) in the presence of 1 µg/ml of ethidium bromide and visualized under UV-Illumination (SynGene, Gene Genius Bio Imaging System, UK). The amplified DNA fragments from tuberculosis patients, household contacts and healthy volunteers were purified from the gel using the Gel extraction kit (Promega) according to the manufacturer's instructions, and subjected to double stranded DNA sequencing to detect the polymorphism. The nucleotide sequences were analyzed using GeneDoc (Version 2.6.002 Nicholas and Nicholas 1997).

### *In silico* analysis

The amplified −591 to +55 region of the IL*-12Rβ2* promoter fragment was analyzed for the presence of transcription factor binding motifs, (http://motif.genome.jp).

### Promoter–reporter constructs

Using DNA with CC genotype as template the −591 to +55 5′ flanking region of the *IL-12Rβ2* gene was PCR amplified utilizing primers −591F and +55R [9] (Table 2) and high fidelity proof reading enzyme (Pfu DNA polymerase, MBI). To facilitate cloning, SacI and NheI sites were incorporated in the forward and reverse amplimers. The purified 646 bp amplicon was cloned upstream to the luciferase reporter gene in pGL-3-Basic to generate pGIL-12Rb2-C construct. To examine the role of C to T polymorphism in the cognate AP-4 binding motif (GAGCTG) present in the pGIL-12Rb2-C construct, it was changed to GAGTTG by site directed mutagenesis to generate the pGIL-12Rb2-T construct.

**Table 2:** List of primers used in the study.

Assay	Name	Sequence
**Sequencing**	IL-12F-780	5′ - agagcggttttaaggtaatgccca - 3′ (622 bp )^a^
	IL-12R -159	5′ - actcccgtataggtcccgtgtt - 3′
**Cloning**	−591F^b^	5′- gcgc**gagctc**gatatctaaataaaatctct - 3′ (646 bp)
	+55R^c^	5′- agttccctgatg**gctgtc**aaca- 3′
**Mutagenesis**	FTMU	5′- attatgcagagttgccgacccct - 3′
	RTMU	5′- aggggtcggcaactctgcataat - 3′
**EMSA**	C probe S	5′- attatgcagag**c**tgccgacccctct - 3′
	C probe AS	5′- agaggggtcggca**g**ctctgcataa t-3′
	T probe S	5′- attatgcagag**t**tgccgacccctct - 3′
	T probe AS	5′- agaggggtcggcaactctgcataat -3′
	AP4-S	5′- cacccggtcagctggccctacacc -3′
	AP4- AS	5′- ggtgtagggccagctgaccgggtg -3′
	C/EBP-α S	5′- atgtttttatgtaataaaa -3′
	C/EBP-α AS	5′- ttttattacataaaaacat -3 ′
**Real time Primers**	β-Actin –F	5′- agaggggtcggcaactctgcataat -3′ (101 bp )
	β-Actin –R	5′- atgctatcacctcccctgtgtg - 3′
	IL-12mRNA-F	5′- cctgtatcaatagtgatgaaattc -3′ (103 bp )
	IL-12mRNA-R	5′- tcccttctgtatgcaggataaat -3′
	IL-4RT-F	5′- aacagcctcacagagcagaagac - 3′ (101 bp )
	IL-4RT-R	5′- gccctgcagaaggtttcctt - 3′
	GATA3RT-F	5′- gcgggctctatcacaaaatga -3′ ( 79 bp )
	GATA3RT-R	5′- gctctcctggctgcagac-3′
	IFNαRT- F	5′- gctgaatgacctggaagcctgtg - 3′ (169 bp )
	IFNαRT-R	5′- gatttctgctctgacaacctccc - 3′

a Amplicon size.

b *Sac*I site in bold incorporated in the −591F primer.

c *Nhe*I site in bold incorporated in the +55R primer.

### Site directed mutagenesis

The purified promoter reporter construct (pGIL-12Rb2-C) harboring C nucleotide (wild type allele) at the polymorphic site (−237) was used as a template for the site-directed mutagenesis. For mutagenesis of the −237 polymorphic site, the Quick-change site-directed mutagenesis kit (Stratagene, La Jolla, CA, USA) and the primers FTMU 5′-attatgcagagttgccgacccct-3′ and RTMU 5′-aggggtcggcaactctgcataat- 3′ containing T nucleotide at the −237 position were used. The resulting construct pGIL-12Rb2-T obtained was used for transfection studies after confirming the polymorphism by DNA sequencing.

### Human Cell lines

Jurkat cells (T cell lymphoma, NCCS, India) and THP-1 (monocyte cell line, ATCC, USA) were cultured in RPMI-1640 (GIBCO) supplemented with 10% heat inactivated fetal bovine serum (GIBCO) and antibiotics, (100 u/ml penicillin, 75 µg/ml streptomycin, 50 µg/ml gentamycin) in a humidified, 5% CO2 air atmosphere incubator, at 37°C. The U-87MG (glioblastoma, ATCC, USA) cells were grown under identical conditions with supplements in DMEM (Life Technologies).

### Transfection and Luciferase Assays

1×10^6^ Jurkat/THP-1 cells/ml suspended in plain RPMI media were co-transfected with the promoter reporter constructs (2 µg) and renilla expression vector, pRL-TK (0.5 µg) using 7 µl of Lipofectamine (Invitrogen). Cells transfected with pGL3-Control vector (SV-40 promoter, Promega) and promoter less vector pGL3-Basic, served as positive, and negative controls, respectively. The U-87MG cells were transfected as described [44]. Thirty-six hours later, transfected cells were stimulated with PHA (1 µg/ml) and PMA (50 ng/ml, Sigma) for five hours. Then the stimulated cells were washed with cold PBS, lysed (Passive Lysis Buffer, Promega) and centrifuged (10,500 g) for 10 min at 4°C. Firefly and renilla luciferase activities in supernates were estimated according to manufacturer's protocol, (Dual Glo Luciferase assay system, Promega; Sirius-Single Tube Luminometer, Berthold Detection Systems, Australia). Transfection efficiency was normalized based on the renilla luciferase activity. The normalized firefly luciferase activity was expressed as fold increase over pGL3–Basic.

### Electrophoretic mobility shift assays (EMSA)

The change in the binding of the AP-4 transcription factor to its motif with C/T being present was evaluated using EMSA. Sense and anti-sense oligonucleotides (Table 2) extending from −248 to −224 containing the wild type (GAGCTG) or polymorphic (GAGTTG) AP-4 binding motifs, were annealed and end labeled ([γ-32 P] ATP, 3000 Ci mmol-1, BRIT, India), using T4 polynucleotide kinase, (Promega). The purified labeled probes were quantified. Nuclear extracts were prepared from stimulated Jurkat T cells as described [45]. The protein content was estimated by Bradford assay.

The nuclear extract containing 8.0 µg of protein was incubated, with 0.5 µg non-specific oligo poly (dI-dC).poly (dI-dC) (Sigma) in binding buffer (20% glycerol, 5 mM MgCl2, 2.5 mM EDTA, 2.5 mM DTT, 250 mM NaCl, 50 mM Tris–HCl, pH 7.5) for 10 min at 4°C in a final volume of 20 µl. Radiolabeled ds-DNA fragments (40,000 cpm) in the presence or absence of unlabeled probes were added to this mixture and incubated at 25°C for 25 min. The DNA–protein complexes were resolved on 5% non-denaturing polyacrylamide gel (0.5× TBE buffer at 4°C at 160 V), autoradiography using a Kodak intensifying screen, scanned using the Personal Molecular Imager (Bio-Rad, USA) and densitometric analysis was done (Quantity-One program (Bio-Rad). To determine the specificity, EMSA experiments were performed with the known AP-4 consensus, [46] (positive) and nonspecific (C/EBP-α, negative control) probes, [47].

### Super shift assay

For the super shift analysis, the anti AP-4 antibody (5X, Santacruz Biotechnology, CA, USA) was added to the nuclear extract (8 µg) and incubated overnight at 4°C, Thereafter the labeled AP-4, C & T probes were added and the complexes formed were resolved and autoradiographed. The reaction without antibody and without nuclear lysate was included as control.

### RNA isolation and cDNA synthesis

All RNA extractions were carried out using 1.5 ml blood sample derived from the tuberculosis (N = 28) patients and healthy volunteers (N = 28) using the RNeasy Blood Mini Kit (Qiagen GmbH (Hilden, Germany) according to manufacturer's instructions, with modification of an additional wash with Erythrocyte Lysis buffer (EL buffer) and inclusion of on column DNAse treatment Qiagen GmbH (Hilden, Germany). The RNA was eluted in distilled DEPC-treated water and stored at −80°C. The quality and quantity of the RNA was estimated by measuring the OD at 260/ 280 nm and 260 nm using the Nanodrop (ND1000, Nano Drop Technologies Inc. Wilmington, DE, USA). For reverse transcription, DNA-free RNA (500 ng) from each sample was mixed with 2 µM of Oligo-dT (100 µM stock) and DEPC-treated water and denatured at 70°C for 10 min and immediately chilled, in an ice bath. To this denatured mixture, reaction cocktail containing, 1× first strand buffer (5× buffer), 1 mM DTT (10 mM stock), 1 mM dNTPs (10 mM stock) and 8 U of RNAsin was added. The mixture was incubated at 25°C for 10 min followed by the addition of 200 U of Reverse Transcriptase (Promega). The cocktail was incubated at 37°C for 90 min, after which the Reverse Transcriptase was inactivated at 70°C for 10 min and the resulting cDNA aliquots were stored at −80°C.

### Real time PCR

The cDNA obtained was subjected to Real Time PCR analysis using primer pairs for the *IL-12Rβ2* (IL-12mRNA-F and IL-12mRNA-R), IL-4 (IL-4RTF and IL-4RTR) [48], IFN-α ( IFN-αRTF and IFN-αRTR), GATA-3 (GATA-3F and GATA-3R), [49] and for *β-Actin* (β Actin-F & β -Actin–R) genes respectively, (Table 2). Intron flanking primers were designed for the *IL-12Rβ2, IL-4 and GATA- 3* genes. PCR master reaction mix containing Power SYBR Premix Ex Taq (Takara, Shiga, Japan), 0.5 µM concentration of each primer (for β-actin, GATA-3, IFN-α) and 0.9 µM for IL-4. 100 ng of cDNA of each sample in optically clear PCR tubes was set up. Real Time detection of transcripts was carried out in MyIQ cycler (Bio-Rad, USA) using SYBR Green. The cycling parameters for IL-12Rβ2, IFN-α and β-actin was as follows: denaturation at 95°C for 5 min; 40 cycles each of denaturation at 95°C for 30 sec, annealing for 30 sec at 60°C and extension at 72°C for 45 sec. For IL-4 and GATA-3 the parameters were identical except for the annealing and extension temperatures which was 60°C and 66°C for 1 min in case of GATA-3 and IL-4 respectively. The Ct values obtained were used for further data interpretation. The melt curve was generated with a ramp rate of 2% in order to enable the generation of the melt curve.

Assessment of the IL-12Rβ2 mRNA expression in tuberculosis patients was carried out. Equivalent numbers of healthy volunteers were also included, in order to obtain the relative quantification of the mRNA expression of *IL-12Rβ2* gene. For the same purpose, the normalized expression was calculated from the threshold cycle values (Ct) normalized to β-actin Ct values (ΔCt = CtIL-12Rβ2−Ctβ-actin) Then ΔΔCt for each target was calculated as ΔCt patient – ΔCt healthy volunteers. The normalized expression of *IL-12Rβ2* gene relative to healthy volunteers was calculated as 2- Δ ΔCt, as described [15]. The graph was plotted relative to the mRNA expression of the miscellaneous healthy laboratory volunteers. For group analysis for the expression of the IL-4, IFN-α and the GATA-3 the 2∧(−ΔCt) method as described was used, [50], [51].

### Statistical analysis

STATA 9.2 was used (Statacorp 2003, College Station, USA). Student's *t* test with unequal variances was used to compare the transcriptional efficiency. Non parametric Mann-Whitney test was used to compare the differences in the mRNA expression between the study groups using Prism 4.03 (Graph Pad Software Inc., San Diego, CA). Fisher's exact test was applied to establish the relationship between the polymorphism in the promoter region and its activity and/mRNA expression.
